# *Staphylococcus aureus *nasal carriage and microbiome composition among medical students from Colombia: a cross-sectional study

**DOI:** 10.12688/f1000research.22035.2

**Published:** 2020-04-21

**Authors:** Niradiz Reyes, Oscar Montes, Stephanie Figueroa, Raj Tiwari, Christopher C. Sollecito, Rebecca Emmerich, Mykhaylo Usyk, Jan Geliebter, Robert D. Burk

**Affiliations:** 1 School of Medicine. Department of Basic Sciences. Research group of Genetics and Molecular Biology, University of Cartagena, Cartagena, Bolivar, 130001, Colombia; 2 Department of Microbiology and Immunology, New York Medical College, Valhalla, New York, 10595, USA; 3 Department of Pediatrics, Albert Einstein College of Medicine, Bronx, New York, 10461, USA; 4 Departments of Microbiology & Immunology; Epidemiology & Public Health; and, Obstetrics, Gynecology & Women’s Health., Albert Einstein College of Medicine, Bronx, New York, 10461, USA

**Keywords:** Microbiome, mycobiome, microbiota, Staphylococcus aureus, bacterial communities.

## Abstract

**Background: **The anterior nares are the main ecological niche for *Staphylococcus aureus*, an important commensal and opportunistic pathogen. Medical students are frequently colonized by a variety of pathogens. Microbial interactions in the human nose can prevent or favor colonization by pathogens, and individuals colonized by pathogens have increased risk of infection and are the source of transmission to other community members or susceptible individuals. According to recent studies, the microbiome from several anatomic areas of healthy individuals varies across different ethnicities. Although previous studies analyzed the nasal microbiome in association with *S. aureus* carriage, those studies did not provide information regarding ethnicity of participants. Our aim was to assess *S. aureus* nasal carriage patterns and prevalence among medical students from Colombia, a country of Hispanic origin, and to investigate possible associations of colonization and nasal microbiome composition (bacterial and fungal) in a subgroup of students with known *S. aureus* carriage patterns.

**Methods: **Nasal swabs from second-year medical students were used to determine prevalence and patterns of *S. aureus* nasal carriage. Based on microbiological results, we assigned participants into one of three patterns of *S. aureus* colonization: *persistent, intermittent*, and *non-carrier*. Then, we evaluated the composition of nasal microbial communities (bacterial and fungal) in 5 individuals from each carriage category using 16S rRNA and Internal-Transcribed-Spacer sequencing.

**Results: **Prevalence of *S. aureus* nasal carriage among medical students was 28%. Carriage of methicillin-resistant strains was 8.4% and of methicillin-sensitive strains was 19.6%. We identified 19.6% persistent carriers, 17.5% intermittent carriers, and 62.9% non-carriers.

**Conclusions:** Analysis of nasal microbiome found that bacterial and fungal diversity was higher in individuals colonized by *S. aureus* than in non-carriers; however, the difference among the three groups was non-significant. We confirmed that fungi were present within the healthy anterior nares at substantial biomass and richness.

## Introduction

The anterior nares represent the main ecological niche for *Staphylococcus aureus*, a bacterium that behaves both as commensal and opportunistic pathogen ^1^. Asymptomatic carriage of *S. aureus* in healthy individuals has high prevalence, especially in children, young adults, and healthcare workers, including medical students ^2– 4^. Due to their frequent contact with the general community and healthcare environment, medical students commonly encounter a variety of pathogens that may colonize them. Individuals colonized by pathogens have increased risk of infection, but also they become a source of transmission to other community members or susceptible patients ^1, 5– 7^.

Microorganisms residing in a particular anatomical site engage in complex interactions that prevent or favor colonization by pathogens ^8^. The complete collection of microbes colonizing the anatomical areas of the human body constitute the microbiota, which includes bacteria, archaea, viruses, and fungi, intertwined in a complex network of interactions among them and the host ^9^. The genes and genomes harbored by these microbial communities make up the human microbiome ^10, 11^. According to recent studies, the microbiome from several anatomic areas of healthy individuals varies across different ethnicities ^12– 15^. Although previous studies have analyzed the nasal microbiome in association with *S. aureus* carriage, these studies have not provided information on the ethnicity of participants, or have used individuals from different ethnic populations ^16, 17^. Differences in microbiome composition linked to ethnic background highlight the need to consider and potentially account for ethnic diversity in microbiome research ^12^. This study sought to determine the nasal prevalence and long-term carrier patterns of *S. aureus* in second-year medical students with Hispanic background. Additionally, we aimed to explore biodiversity of the nasal microbiome (bacterial and fungal) and possible differential abundance of specific taxa among the three categories of *S. aureus* long-term carriage.

## Methods

### Study design and population

The Ethics Review Boards of the University of Cartagena (Approval #280313) and New York Medical College approved this study (Protocol # 12697; IRB ID: 12697). The study was conducted between January and June of 2018 and enrolled second-year medical students from University of Cartagena, Colombia, who had not yet engaged in clinical rotations. To prevent sampling bias, we aimed to enroll the complete population of second-year medical students of our institution. Students were recruited via fliers and lecturer announcements. Those who agreed to participate signed an informed consent and completed a written questionnaire on demographics and medical history before each nasal swab sampling. Exclusion criteria were recent infections, allergies and other non-infectious pathologies, smoking habits, antibiotic usage in the previous three months, surgeries and hospitalizations in the previous six months. In total 143 out of 158 second-year medical students completed the study. Thus, we enrolled 90.5% of the total population of second-year medical students in our institution in this study. We followed the STROBE cross sectional reporting guidelines ^18^.

### Specimen collection, prevalence of colonization and carriage categories

Nasal swabs were obtained from both nostrils by a trained individual, inoculated into Stuart transport medium (OXOID, England), transported to the microbiology laboratory and processed within 8–18 hours according to described protocols ^6^. *S. aureus* was identified based on colony morphology, Gram-stain, catalase-test, tube coagulase-test, and latex agglutination-test. Genomic DNA was obtained from each isolate with Wizard ^®^ Genomic DNA Purification Kit (Promega, USA) and *S. aureus* molecular confirmation and methicillin-resistance were assessed by PCR-amplification using specific primers for *nuc* and *mec*A genes, respectively. Detailed protocols for all these methods, including primer sequences, have been previously described ^6^. To determine prevalence of *S. aureus* nasal carriage, each participant was classified either as carrier or non-carrier based on laboratory results obtained from the first nasal swab survey. To establish *S. aureus* long-term carriage categories, four additional consecutive nasal swabs were obtained from each participant, in three-week intervals. According to definitions proposed by *Kluytmas et al*. ^19^, participants that yielded five negative cultures for *S. aureus* were classified as non-carriers; those yielding one to three positive cultures were classified as intermittent carriers; and those yielding four or five positive cultures were classified as persistent carriers.

### Nasal microbiome (bacterial and fungal) analysis

This study aimed to describe the microbiome composition in a small group of second-year medical students with a known *S. aureus* carriage status. At the end of the study, 15 participants with known *S. aureus* long-term carriage status (5 non-carriers, 5 intermittent-carriers, and 5 persistent-carriers) were randomly selected from the cohort of 143 participants. The 15 selected participants provided an additional nasal swab that was stored at room temperature in Amies-Transport-Medium with Charcoal (Copan Diagnostics, Inc., Murrieta, CA), and sent to the laboratory of Robert D. Burk at Albert Einstein College of Medicine (AECOM) for microbiome analysis. Sample processing was performed according to protocols described by Usyk *et al*. ^20^. The time between collection and processing of samples was around 8 days.

### 16S rRNA gene and Internal Transcribed Spacer (ITS) PCR-amplification

The V4 hypervariable region of 16S rRNA gene was amplified using primers 16SV4_515F (GTGYCAGCMGCCGCGGTA) and 16SV4_806R (GGACTACHVGGGTWTCTAAT), with a unique 12-bp barcode Golay-barcoding ^21, 22^. PCR conditions were: initial 5min denaturation at 95°C, followed by 15-cycles of 95°C for 1min, 55°C for 1min, and 68°C for 1min, and final extension for 10min at 68°C. For the fungal component of microbiome, barcoded amplicons were generated covering the ITS gene region using ITS1-30F/ITS1-217R primer pair, as previously described by Usyk *et al*. ^20^ (ITS1-30F: 5’-GTCCCTGCCCTTTGTACACA-3’ and ITS1-217R: 5’-TTTCGCTGCGTTCTTCATCG-3’). PCR conditions were: 3min initial denaturation at 95°C, followed by 35-cycles of 95°C for 30sec, 55°C for 30sec, and 68°C for 2min, followed by final extension at 68°C for 10min. PCR reagents were obtained from Affymetrix (Affymetrix, Santa Clara, CA). PCR reactions were run in GeneAmp PCR-System 9700 (Applied Biosystems).

### High-throughput sequencing

PCR products were purified using QIAquick Gel Extraction Kit (QIAGEN) and quantified using Qubit ^TM^ dsDNA High-Sensitivity Assay kit (Life Technologies). Next-generation sequencing library preparation was performed using KAPA-LTP library preparation kit (KAPA Biosystems, Wilmington, MA). Size integrity of isolated amplicons was validated with 2100 Bioanalyzer (Agilent-Technologies, Santa Clara, CA). High-throughput sequencing of libraries was performed using Illumina HiSeq2500 Sequencing System (Illumina, San Diego, CA) with a 2×250-bp paired-end read kit at the Genomics Core Facility of AECOM.

### Bacterial microbiome bioinformatics analysis

Illumina reads were pre-processed to remove bases that fell below PHRED quality score of 25 using PRINSEQ ^23^. Processed reads were de-multiplexed using sample specific barcode combinations with Novobarcode V1.00. This can also be performed with deML, a program freely available for use under the GPL license. Paired-end reads were merged using free open source PANDAseq v1.20 with default settings ^24^. OTU-clustering and quality filtering was performed using the Quantitative Insights Into Microbial Ecology ( QIIME v1.9) software package ^25^. Removal of sequencing noise and sequence chimeras was done with USEARCH v8.0 ^26^. Sequences were de-multiplexed and clustered into operational taxonomic units (OTUs) with 97% minimum cluster similarity using UCLUST ^26^. Sequences were assigned using UCLUST with Greengenes 13.8 microbial database ^27^. The resulting **BIOM** table was rarefied to 29,000 reads/sample and statistical analyses were performed after collapsing OTUs at genus level.

### Fungal microbiome bioinformatics analysis

Sequence reads were processed using open-reference OTU-picking with QIIME v1.9 against the targeted host-associated fungi ITS database (THF1) ^28^ for the reference-based clustering component. VSEARCH v1.4.0 ^29^ was used to de-replicate reads, cluster reads into OTUs and remove chimeric sequences. OTU-clustering threshold was set at 99% sequence identity to account for fungal heterogeneity. Sequence de-replication and chimera removal were performed using QIIME quality-control protocol. Representative sequences for each OTU-cluster were chosen based on sequence abundance. BLAST was used to assign taxonomy using the UNITE database ^30^. The default behavior of BLAST in QIIME was changed to minimum of 99% sequence identity for taxonomic assignment. Data were processed in R v3.3.1 ^31^. QIIME outputs were imported into R using *phyloseq package v1.22.3* ^32^ and further processed with *vegan v2.5-3* ^33^, *coin v1* ^34^, and *reshape2* ^35^. Data visualization was performed using *ggplot2* ^36^.

### Alpha/Beta diversity analysis of 16S rDNA-V4 and ITS sequences

Statistical analyses were performed to assess differences in OTU distribution and abundance between samples and groups. Microbial diversity for bacterial and fungal communities was evaluated within samples (α-diversity) or between samples (β-diversity) using QIIME. Rarefaction to subsampling depth of 29,000 reads/sample or 9,000 reads/sample, for bacteria or fungi respectively, and 5 iterations were performed on all samples to standardize the sequencing effort. Alpha-diversity was measured with Chao1 (richness) and Shannon entropy (OTU-based diversity) index. Beta-diversity was calculated using Bray-Curtis dissimilarity coefficient. To test for dissimilarities in the microbial composition between *S. aureus* carrier groups, non-metric multidimensional scaling (NMDS) was performed with Bray-Curtis dissimilarity.

## Results

### *S. aureus* nasal carriage

The first nasal swab isolated *S. aureus* from 40 out of 143 participants, for a prevalence of 28%. Methicillin-resistant *S. aureus* (MRSA) was carried by 12 participants (8.4%) and methicillin-sensitive *S. aureus* (MSSA) by 28 (19.6%). The longitudinal study identified 28 (19.6%) persistent carriers, 25 (17.5%) intermittent carriers, and 90 (62.9%) non-carriers. MRSA strains were isolated from 6 persistent carriers and 6 intermittent carriers. Table 1 lists the characteristics of students used in the microbiome study. *Underlying data:* Table S1 ^37^ lists the main results obtained from the complete study population.

**Table 1.** Characteristics of participants of the microbiome study.

Participant code	Long-term carriage category	Age	Gender
JG06	Intermitent	22	M
JG07	Intermitent	19	F
JG08	Intermitent	19	F
JG09	Intermitent	20	M
JG10	Intermitent	18	M
JG11	Non carrier	18	F
JG12	Non carrier	21	F
JG13	Non carrier	18	F
JG14	Non carrier	28	M
JG15	Non carrier	19	M
JG01	Persistent	22	F
JG02	Persistent	21	F
JG03	Persistent	20	F
JG04	Persistent	20	M
JG05	Persistent	28	M

### Diversity and abundance of resident bacterial and fungal communities

**Figure 1.**
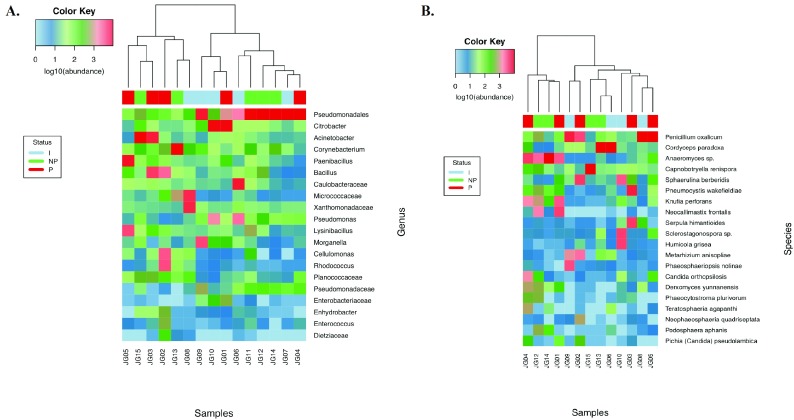
Cluster analysis of bacterial genus **(A)** and fungal species **(B)** compositions in the nares of healthy medical students with a known *S. aureus* carriage pattern. Heatmap was constructed using normalized log10 abundance of each OTU in each sample type. Data are presented only for the 20 most abundant taxa. Colored bar above heatmap indicates *S. aureus* carriage status. P: *persistent (red),* I: *intermittent (blue),* NP: *non-carrier (green).*

Figure 1 shows clustering analysis of bacterial genus (A) and fungal species (B) compositions of nasal specimens from the cohort of 15 healthy medical students with known *S. aureus* carriage patterns. Bacterial microbiome analysis, sequencing, quality filtering and mapping resulted in 1,424,972 mapped V4-region sequences, ranging between 29,971-217,540 copies per sample (average 120,062; SD = 53,027), corresponding to 600 OTUs. We identified 57 of the 600 OTUs (9.5%) at the genus level, while the remaining OTUs mapped to unclassified genera (70.8%) or upper taxonomic groups (96.8%). Three phyla were identified, *Proteobacteria* being the most abundant (78.3% of 16S rRNA sequences) and the most diverse (42.2% of all identified OTUs), followed by *Firmicutes* and *Actinobacteria*. In general, the nasal bacterial microbiome was dominated by the Class *Gammaproteobacteria* (order *Pseudomonadales* and genera *Citrobacter* and *Acinetobacter*), which is consistent with a 2016-review by Lee *et al.* ^38^. Table 2 shows the predominant bacterial genera isolated from the nostrils of healthy medical students.

**Table 2.** Predominant bacterial genera identified from the nostrils of healthy medical students.

Genus	Order	Phylum	Relative abundance (%)
*Citrobacter*	Enterobacteriales	Proteobacteria	10.3%
*Acinetobacter*	Pseudomonadales	Proteobacteria	9.2%
*Corynebacterium*	Corynebacteriales	Actinobacteria	7.2%
*Paenibacillus*	Bacillales	Firmicutes	4.7%
*Bacillus*	Bacillales	Firmicutes	4.2%
*Pseudomonas*	Pseudomonadales	Proteobacteria	3.0%
*Lysinibacillus*	Bacillales	Firmicutes	2.7%
*Morganella*	Enterobacteriales	Proteobacteria	2.4%
*Rhodococcus*	Actinomycetales	Actinobacteria	2.0%
*Cellulomonas*	Actinomycetales	Actinobacteria	1.9%

**Figure 2.**
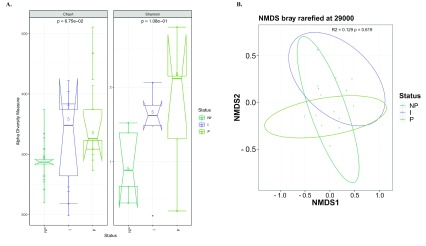
Alpha and beta diversity of bacterial composition of nasal samples. **(A)** Alpha-diversity, measured by Chao1 and Shannon diversity Index, is plotted for individuals with different *S. aureus* carrier status: non-carriers (NP, dark green), intermittent carriers (I, purple) and persistent carriers (P, light green). The Chao1 index (left panel) and Shannon index (right panel) were computed for all 15 subjects. The line inside the box represents the median, while the whiskers represent the lowest and highest values within the 1.5 interquartile range (IQR). Statistical testing showed no significant differences among the groups: Chao1 *p* = 0.0675; Shannon *p* =0.108. **(B)** Comparison of beta-diversity of bacterial composition between *S. aureus* carriage groups with NMDS ordinance calculated from Bray–Curtis distance estimation. *I: Intermittent, NP: non-carrier, P: persistent.*

**Figure 3.**
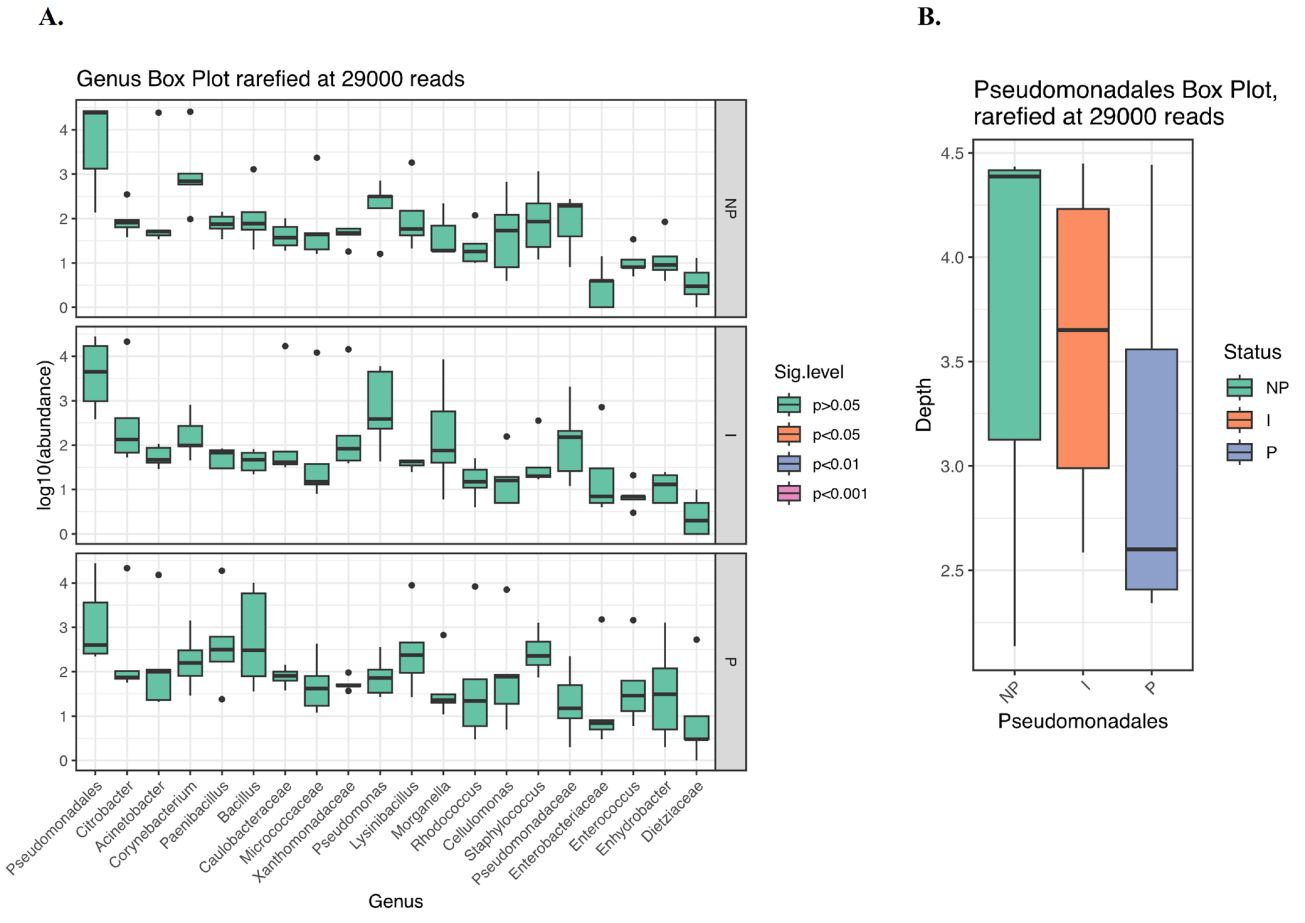
Boxplots representing relative abundance analysis of the bacterial taxa identified in nasal samples of individual from the different *S. aureus* carrier groups. **(A)** Differential abundance at the genus level. **(B)** Differential abundance for the order *Pseudomonadales*. Taxa with minimum median abundance of 1% were used for the comparison. ** There was non-significant difference in abundance among the three carrier groups ( *p>0.05, Kruskal–Wallis test*). *NP: non-carrier, I: Intermittent, P: persistent.*

Alpha-diversity analysis found that bacterial diversity was greater in the persistent group followed by the intermittent and then by the non-carriers, as shown in Figure 2A. However, the difference in diversity among the three carrier groups was statistically non-significant. Beta-diversity analysis of bacterial communities showed non-significant separation of groups ( Figure 2B). Analysis of differential abundance identified that order *Pseudomonadales* was more abundant in non-carriers followed by intermittent and persistent categories ( Figure 3), a finding that may have implications regarding microbial antagonism. However, this trend did not reach statistical significance ( *p>0.05, Kruskal–Wallis test*).

For fungal microbiome analysis, sequencing, quality filtering and mapping resulted in 4,274,743 mapped ITS-region sequences, ranging between 9,543-743,278 copies per sample (average 284,983; SD = 237,339), corresponding to 8,346 fungal OTUs (average 556; SD = 333) ( Table 3). We were able to classify 4,453 of the 8,346 OTUs (53.3%) down to species level. Out of the seven recognized major phylum of fungi, three phyla were identified in the nostrils, being *Ascomycota* the most abundant (90.8% of fungal sequences) and the most diverse (88.7% of all identified OTUs), followed by *Basidiomycota* and *Neocallimastigomycota* (containing anaerobic fungi). In general, the nasal mycobiome was dominated by species of the phylum *Ascomycota,* which is consistent with a 2013-publication by Findley, *et al*. ^39^. Table 4 shows the predominant fungi species identified from the nostrils of healthy medical students. The most frequent fungal species detected was *Penicillium oxalicum*, representing 19.0% of the total 4,274,743 sequence reads in the chimera‐filtered OTU table. *Malassezia restricta* was the second most frequent fungus (12.2% of all sequences). 4.4% of all sequences were unassigned sequences, thought to represent non-fungal contamination.

**Table 3.** Number of fugal sequences and OTUs from medical students with known *Staphylococcus aureus* carriage status.

Sample	Carriage Category	^a^OTUs Clustered	^b^Fungal sequences
JG11	Non carrier	125	9,543
JG12	Non carrier	245	25,175
JG13	Non carrier	727	743,278
JG14	Non carrier	381	160,273
JG15	Non carrier	720	621,267
**Average: 440 311,907**			
JG06	Intermittent	491	265,710
JG07	Intermittent	154	14,612
JG08	Intermittent	740	341,059
JG09	Intermittent	578	289,560
JG10	Intermittent	502	366,262
**Average: 493 255,441**			
JG01	Persistent	272	57,915
JG02	Persistent	855	499,815
JG03	Persistent	725	281,963
JG04	Persistent	404	48,946
JG05	Persistent	1427	549,365
**Average: 737 287,601**			
**Total in the cohort: 8,346 4,274,743**			

**Table 4.** Predominant fungi species identified from the nostrils of healthy medical students.

Species	Order	Phylum	Relative abundance (%)
*Penicillium oxalicum*	Eurotiales	Ascomycota	19.0%
*Malassezia_restricta*	Malasseziales	Basidiomycota	12.2%
*Capnobotryella_renispora*	Capnodiales	Ascomycota	7.5%
*Nectria_cinnabaarina*	Hypocreales	Ascomycota	7.4%
*Calonectria_asiatica*	Hypocreales	Ascomycota	6.6%
*Cladosporium_phaenocomae*	Capnodiales	Ascomycota	5.2%
*Lipomyces_doorenjongii*	Saccharomycetales	Ascomycota	4.0%
*Knufia_perforans*	Chaetothyriales	Ascomycota	3.8%
*Rhodosporidium_lusitaniae*	Ustilaginales	Basidiomycota	3.7%
*Serpula himantioides*	Boletales	Basidiomycota	3.6%

**Figure 4.**
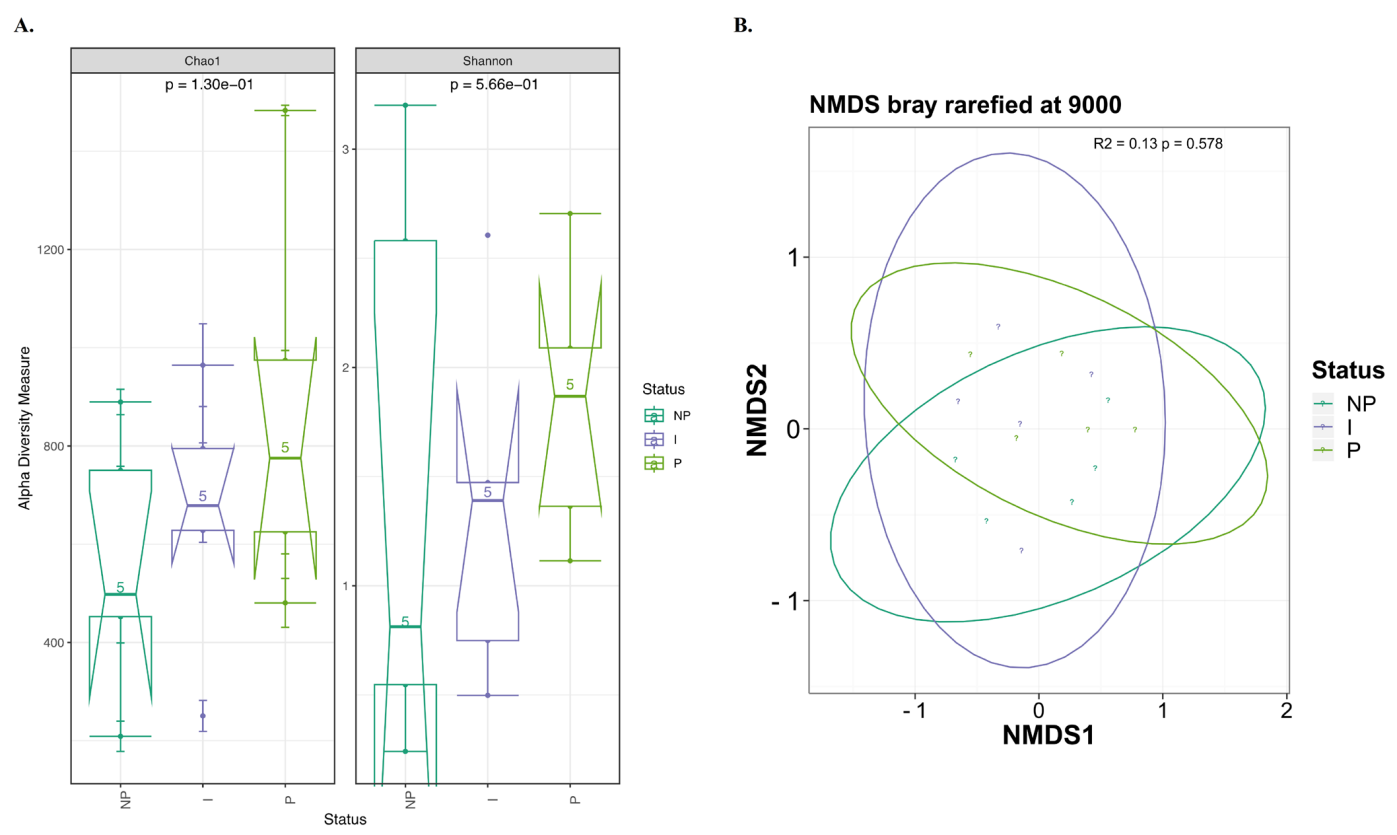
Alpha and beta diversity of fungal composition of nasal samples. **(A)** Alpha-diversity, measured by Chao1 and Shannon diversity Index is plotted for individuals with different *S. aureus* carrier status: non-carriers (NP, dark green), intermittent carriers (I, purple) and persistent carriers (P, light green). The Chao1 index (left panel) and Shannon index (right panel) were computed for 15 subjects. The line inside the box represents the median, while the whiskers represent the lowest and highest values within the 1.5 interquartile range (IQR). Statistical testing showed no significant differences among the groups: Chao1 *p* = 0.13; ** Shannon *p* = 0.566. **(B)** Comparison of beta-diversity of fungal composition between *S. aureus* carriage groups with NMDS ordinance calculated from Bray–Curtis distance estimation. *I: Intermittent, NP: non-carrier, P: persistent.*

Based on the identified OTUs and number of sequences in this study, we report a substantial diversity and amount of fungal biomass in the anterior nares of this group of medical students with Colombian ethnicity. Consistent with the 16S analysis, alpha-diversity of fungal communities showed that fungal diversity was greater in the persistent group followed by the intermittent and then by the non-carrier groups. However, this trend did not reach statistical significance ( Figure 4A). Beta-diversity analysis of fungal communities showed non-significant separation of groups ( Figure 4B).

**Figure 5.**
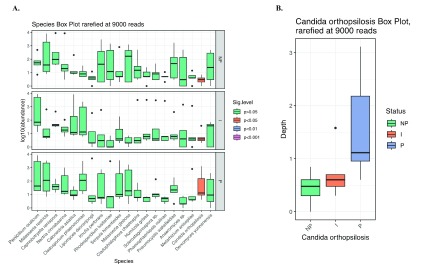
Boxplots representing relative abundance analysis of the fungal taxa identified in nasal samples of individual from the different *S. aureus* carrier groups. **(A)** Differential abundance at the species level. **(B)** Differential abundance for the species *Candida orthopsilosis* showed a significant difference in abundance between the persistent and non-carrier groups ( *p<0.05, Kruskal-Wallis test*). Taxa with minimum median abundance of 1% were used for the comparison. *NP: non-carrier, I: Intermittent, P: persistent.*

Analysis of differential abundance showed increased abundance for several fungi species in the persistent group compared to the intermittent and non-carriers ( Figure 5A). However, the species *Candida orthopsilosis* was the only one with significant difference in abundance between the persistent and non-carrier groups ( *p<0.05, Kruskal-Wallis test*) ( Figure 5B).

## Discussion

Prevalence of *S. aureus* nasal carriage was 28%, which is consistent with other studies ^7^. We also found that 37.1% of second-year medical students carried *S. aureus* in their nares, persistently or intermittently. The distinction in carriage category is important, as persistent carriers are at higher risk of developing active autoinfection than intermittent and non-carriers ^40– 43^. MRSA was carried by 8.4% of participants, which represents an important increase from the 1.6% MRSA carriage that we previously reported for our institution in 2012 ^6^.

We sought to analyze both bacterial and fungal composition in individuals with a known long-term nasal carriage pattern for *S. aureus.* We found that nasal bacterial microbiome had low diversity at the phylum level, with three dominating phyla: Proteobacteria, Firmicutes, and Actinobacteria ^44, 45^. The top five most abundant genera were *Citrobacter, Acinetobacter, Corynebacterium, Paenibacillus* and *Bacillus,* all of which contain pathogenic species, evidencing the potential of the anterior nares as reservoir for pathogens ^46– 48^. An interesting finding was that abundance of the genus *Staphylococcus* in the nares was generally low, even in nasal carriers of *S. aureus*. The estimated bacterial richness (# of species) found in our study is consistent with a former study that reported an estimate of 2,264 species in the anterior nares based on V3-V5 16S rRNA sequencing ^49^. Recent studies suggest that composition of the nasal microbiota greatly influences *S. aureus* nasal colonization ^50, 51^. However, the mechanisms used by the nasal microbiota to antagonize *S. aureus* colonization are not completely understood ^46^. Our results are in concordance with those from the Human Microbiome Project in the sense that microbiome composition varies by anatomical site and that interpersonal variation is significant ^52, 53^.

Recently, researchers started to focus on the fungal component of the microbiome, revealing the remarkable diversity of the human mycobiome. Fungi were detected at varying abundance in our three carriage groups. The most abundant fungus identified was *Penicillium oxalicum*, a common environmental fungi that has been recently identified as a cause of invasive mycosis in immunocompromised patients ^54^. Although not statistically significant, we identified a trend towards higher richness and evenness of both bacteria and fungi in the persistent group compared to the intermittent and non-carrier groups. Other studies have suggested that a more diverse microbiota may be associated with resistance to colonization by pathogens; however, we did not observe this phenomenon in our study. Instead, our study found that a more diverse bacterial and fungal microbiome in the anterior nares seems to favor *S. aureus* carriage. A similar finding was reported for the pathogen *Streptococcus pneumoniae*, where a more diverse nasopharyngeal microbiome appeared to facilitate pneumococcal carriage in this human niche ^55^.

We could not evaluate completely the involvement of specific OTUs in *S. aureus* carriage due to the small sample size. However, we could identify that the order Pseudomonadales was enriched in non-carriers and that the fungi species *Candida orthopsilosis* was significantly enriched in the persistent group. These results may suggest that some species in the Pseudomonadales antagonize long-term colonization by *S. aureus* or that *S. aureus* colonization may impact the composition of the underlying bacterial communities in the nares, displacing other microbial communities, as has been proposed by others ^16^. Additional studies are also required to determine whether the presence of *Candida orthopsilosis* in the nose favor the long-term *S. aureus* colonization of human nares. Limitations of this study were that microbiome composition was analyzed in a small set of samples and that stability of the nasal microbiome over time was not analyzed. Since subtle but significant differences in taxonomic composition between different ethnicities have been previously reported ^12^, further studies with larger sample size and defined ethnic background are required to identify the interactions between specific members of the resident microbiota that favor or antagonize the colonization process of the bacterium *S. aureus* in the anterior nares of specific ethnic groups of the human population. This study is the first to analyze simultaneously the bacterial and fungal communities in the nostrils of healthy medical students with a Hispanic/Latino background, and their association with *S. aureus* nasal colonization.

## Data availability

### Underlying data

Nasal Microbiome. Raw sequencing data of the nasal microbiome of a set of medical school students, Accession number, PRJNA600228: https://www.ncbi.nlm.nih.gov/bioproject/600228.

Open Science Framework: Nasal Microbiome of Medical Students UdeC. https://doi.org/10.17605/OSF.IO/UDNWA ^37^.

This project contains the following underlying data:

- Table S1: Data for complete study population
- JG_16S_OTU_L6: Bacterial species found for microbiome study of 15 participants.
- JG_ITS_OTU_L7: Fungal species found for microbiome study of 15 participants.

Data are available under the terms of the Creative Commons Zero "No rights reserved" data waiver (CC0 1.0 Public domain dedication).
