# Supplementary material for: Staphylococcus aureus nasal carriage and microbiome composition among medical students from Colombia: a cross-sectional study
Source: F1000Res. 2020 Apr 21;9:78. Originally published 2020 Feb 3. [Version 2] doi: 10.12688/f1000research.22035.2 (PMC7156025; doi:10.12688/f1000research.22035.2)
Supplement: Peer review history

# Peer review: Reviewer response for version 1

**DOI:** 10.5256/f1000research.24300.r61250

**Published:** 2020-04-08

**Authors:** Anne-Sofie Furberg

**Affiliations:** 1 Molde University College, Molde, Norway; 2 University Hospital of North Norway, Tromsø, Norway

The authors aim to assess the prevalence of *S. aureus* nasal carriage and *S. aureus* nasal carriage phenotypes among medical students in Colombia, and to examine whether *S. aureus* nasal carriage is associated with composition of the nasal microbiome. *S. aureus* nasal carriage among medical students and health care workers may represent a source for transmission of the microbe and infection in susceptible patient groups. *S. aureus* carriage is partly determined by interactions with other members of the microflora. According to the authors, this is the first study analyzing simultaneously the bacterial and fungal communities in the nares of healthy medical students with a Hispanic/Latino background. The study was done among second-year medical students at the same university during a six months period in 2018. The authors collected five repeated nasal swabs for *S. aureus* culturing from 143 students, and an additional nasal swab for microbiome analysis from a random sample of 15 students having different *S. aureus* carriage phenotypes. Using only data from the first set of nasal swabs, they report a prevalence of *S. aureus* carriage of 28%. Using data from all five time points, they report that 20% are persistent carriers. They show that nasal samples from persistent carriers had higher bacterial and fungal diversity than intermittent carriers and non-carriers, however not statistically significant. *S. aureus* carrier groups could not be separated by bacterial or fungal diversity.

 The manuscript is generally well written and the results are easy to read from tables and figures. The classification of *S. aureus* carriage phenotypes by repeated sampling in a relatively large sample is a major strength. The analysis of *S. aureus* carriage in relation to the microbiome is timely and relevant. However, the present study has some limitations that should be addressed.

 Microbiome data was collected at the end of the study among 15 students within the three *S. aureus* carriage phenotype groups. It would be interesting to know more about the stability of the nasal microbiome over time. Did the authors consider taking nasal swabs for microbiome analysis at another time point, e.g. at “baseline” when the first set of swabs for *S. aureus* culturing were taken? Please, consider rephrasing the sentences in Discussion page 9 “Here, a more diverse…microbiome ..seems to favor *S. aureus* carriage.” and “Our results also suggest that Candida species may favor the long-term *S. aureus* colonization of human nares.”, as the design of the present study may not be in line with these interpretations. Please, explain why the microbiome analysis was limited to five students in each group. Did the authors perform any power analysis?

*S. aureus* nasal colonization was assessed at five different time points. Could these data have been used to estimate *S. aureus* prevalence?

**Abstract**: The authors may consider a more consistent use of terminology; e.g. patterns of *S. aureus* nasal carriage or patterns of *S. aureus* colonization. First part of conclusion can be moved to results.

**Introduction**: The first sentence may be modified as the anterior nares are important due to several functions.

**Methods**: Five nasal swab samples for *S. aureus* culturing were collected with three weeks intervals. Please, describe how information about factors defined in exclusion criteria was collected and updated during the minimum 12 weeks period; e.g. recent infections, antibiotic use. Please, describe the procedure for specimen collection in more detail; i.e. how did the authors standardize the collection method, who did the sampling and where? The nasal swab for microbiome analysis was kept at room temperature and transported to New York, USA, for analysis. Please, include information about transport time and stability of the material from collection to laboratory analysis.

**Results**: It would be interesting to know more about the characteristics of the study population for comparison with other studies; e.g. sex, age- and BMI distribution. Results and figures: Please, include “statistically” when referring to the results of statistical tests; i.e. statistically non-significant.

Is the work clearly and accurately presented and does it cite the current literature?

Yes

If applicable, is the statistical analysis and its interpretation appropriate?

Yes

Are all the source data underlying the results available to ensure full reproducibility?

Yes

Is the study design appropriate and is the work technically sound?

Yes

Are the conclusions drawn adequately supported by the results?

Yes

Are sufficient details of methods and analysis provided to allow replication by others?

Partly

Reviewer Expertise:

Infectious disease epidemiology.

I confirm that I have read this submission and believe that I have an appropriate level of expertise to confirm that it is of an acceptable scientific standard.

# Response

**Published:** 2020-04-09

**Authors:** NIRADIZ REYES

**Affiliations:** UNIVERSIDAD DE CARTAGENA, Colombia

The authors are thankful for the Reviewer´s recommendations.

 We greatly value your comments and consider that they have improved the quality of our article.

 We have followed them and modified the article accordingly.

# Peer review: Reviewer response for version 1

**DOI:** 10.5256/f1000research.24300.r59469

**Published:** 2020-03-18

**Authors:** Marcelo Brocchi

**Affiliations:** 1 Tropical Disease Laboratory, Department of Genetics, Evolution, Microbiology and Immunology, Institute of Biology, University of Campinas, Campinas, Brazil

This is an interesting study describing the *Staphylococcus aureus* carrying state among medical students in Colombia. The prevalence of MRSA strains was investigated and the association of the *S. aureus* carrying state with microbiome composition investigated in a minor number of individuals. It was observed that bacterial and fungal diversity was higher in individuals colonized by *S. aureus* than in non-carriers although the differences were non-significant. These results contributed with epidemiological information on *S. aureus* carrier state in Colombian medical students. Maybe, microbiome analysis in a greater number of individuals could reveal a more robust association with *S. aureus* carrier state. However, the data presented suggest some interesting associations that deserve publication. My only suggestion to the authors is the inclusion of a paragraph in the discussion section about microbiome composition and ethnic groups. Are there differences among Hispanic and other ethnic groups regarding microbiome composition and diversity?

Is the work clearly and accurately presented and does it cite the current literature?

Yes

If applicable, is the statistical analysis and its interpretation appropriate?

Yes

Are all the source data underlying the results available to ensure full reproducibility?

Yes

Is the study design appropriate and is the work technically sound?

Yes

Are the conclusions drawn adequately supported by the results?

Yes

Are sufficient details of methods and analysis provided to allow replication by others?

Yes

Reviewer Expertise:

Microbiology and Genetics of Microorganisms.

I confirm that I have read this submission and believe that I have an appropriate level of expertise to confirm that it is of an acceptable scientific standard.

# Response

**Published:** 2020-04-09

**Authors:** NIRADIZ REYES

**Affiliations:** UNIVERSIDAD DE CARTAGENA, Colombia

The authors are thankful for the Reviewer´s recommendations.

 We have followed them and modified the article accordingly.
